# CPO Complete, a novel test for fast, accurate phenotypic detection and classification of carbapenemases

**DOI:** 10.1371/journal.pone.0220586

**Published:** 2019-12-11

**Authors:** Gina K. Thomson, Sameh AbdelGhani, Kenneth S. Thomson

**Affiliations:** 1 University of Louisville Hospital, Microbiology Department, Louisville, Kentucky, United States of America; 2 University of Louisville School of Medicine, Department of Pathology and Laboratory Medicine Louisville, Kentucky, United States of America; 3 Beni-Suef University School of Pharmacy, Department of Microbiology and Immunology, Beni-Suef, Egypt; Instiuto Ramon y Cajal de Investigacion Sanitaria (IRYCIS), SPAIN

## Abstract

Carbapenemase-producing organisms (CPOs) are Gram-negative bacteria that are typically resistant to most or all antibiotics and are responsible for a global pandemic of high mortality. Rapid, accurate detection of CPOs and the classification of their carbapenemases are valuable tools for reducing the mortality of the CPO-associated infections, preventing the spread of CPOs, and optimizing use of new β-lactamase inhibitor combinations such as ceftazidime/avibactam, meropenem/vaborbactam and imipenem/relebactam. The current study evaluated the performance of CPO Complete, a novel, manual, phenotypic carbapenemase detection and classification test. The test was evaluated for sensitivity and specificity against 262 CPO isolates of *Enterobacteriaceae*, *Pseudomonas aeruginosa* and *Acinetobacter baumannii* and 67 non-CPO isolates. It was also evaluated for carbapenemase classification accuracy against 205 CPOs that produced a single carbapenemase class. The test exhibited 100% sensitivity 98.5% specificity for carbapenemase detection within 90 minutes and detected 74.1% of carbapenemases within 10 minutes. In the classification evaluation, 99.0% of carbapenemases were correctly classified for isolates that produced a single carbapenemase. The test is technically simple and has potential for adaptation to automated instruments. With lyophilized kit storage at temperatures up to 38°C, the CPO Complete test has the potential to provide rapid, accurate carbapenemase detection and classification in both limited resource and technologically advanced laboratories.

## Background

Carbapenemase-producing organisms (CPOs) are Gram-negative bacteria that are typically resistant to most or all antibiotics. The infections they cause can be difficult or impossible to treat and constitute a major global health threat of high mortality that has been compared to that of Ebola [1–4]. CPOs threaten to take away the utility of antibiotics to both treat infections and to protect vulnerable patients at risk of infection [3, 5]. The occurrence of totally resistant infections brings back the specter of septic wards in which doctors are helpless and can only observe if their patients recover or die. The drivers of this crisis are the extensive antibiotic resistance that results in failures to provide effective therapy coupled with inadequate infection control.

Rapid CPO detection and carbapenemase classification can be pivotal to reducing mortality. Speed of detection is critical for effective infection control and for prompt initiation of combination antibiotic therapy, which is optimal for serious CPO infections [2, 3, 6–17]. The development of the Carba NP test provided a significant advance for clinical laboratories as it provided rapid carbapenemase detection and high accuracy in tests with isolates of *Enterobacteriaceae* and *P*. *aeruginosa* [18]. The utility of this test has been limited by the instability in solution of its imipenem substrate, the high cost of reference standard imipenem powder [19] and suboptimal accuracy for detection of carbapenemases in *A*. *baumannii* [20]. Its accuracy for testing *A*. *baumannii* has been improved with the advent of a modified form of the test [20]. The Carba NP test is not commercially available but derivatives are marketed e.g. the RAPIDEC^®^ CARBA NP (bioMérieux, La Balme-les-Grottes, France) and the Neo-Rapid CARB Kit^®^ (Rosco Diagnostica A/S, Taastrup, Denmark). Neither the Carba NP test nor its marketed derivative tests classify carbapenemases.

Classification of carbapenemases can optimize appropriate use of new β-lactamase inhibitor combinations such as ceftazidime/avibactam, meropenem/vaborbactam, imipenem/relebactam, aztreonam/avibactam, meropenem/nacubactam, cefepime/zidebactam and cefepime/VNRX-5133 and minimize their overuse. The Carba NP test II is an extension of the Carba NP test that was developed to detect and classify the carbapenemases of *Enterobacteriaceae* and *Pseudomonas* spp. by utilization of tazobactam and EDTA as inhibitors of carbapenemases of classes A and B respectively [21]. Another rapid approach to rapid carbapenemase detection and classification is immunochromatographic (ICA) testing such as the NG-Test Carba 5 (NG, Biotech, Guipry, France). These tests are capable of detecting and classifying single or co-production of a limited range of carbapenemases such as NDM, VIM, KPC, OXA-48-like and IMP types [22]. Compared to the comprehensive target range of the Carba NP-derived tests, the role of the narrow-range ICA tests may be more as a second-line test after broad-spectrum carbapenemase screening based on biochemistry [23].

The CPO Complete test is a manual CPO detection and classification test designed to provide rapid, accurate results in tests with *Enterobacteriaceae*, *P*. *aeruginosa* and *A*. *baumannii*. The current study was designed to assess its speed and accuracy of CPO detection and its potential to classify carbapenemases.

## Materials and methods

### Isolates

Three hundred twenty nine isolates of *Enterobacteriaceae*, *Pseudomonas aeruginosa* and *Acinetobacter baumannii* from two culture collections, that of the University of Louisville and also the Antimicrobial Resistance Isolate Bank of the Centers for Disease Control and Prevention and Food and Drug Administration, were characterized for types of β-lactamase production by PCR, microarray, DNA sequencing, whole genome sequencing, phenotypic and biochemical tests. Those tested in the carbapenemase detection phase of the study included isolates of high diagnostic difficulty. They included 125 isolates producing KPC, NMC-A, IMI, and SME class A carbapenemases; 87 isolates producing NDM, SPM, IMP, VIM and GIM class B carbapenemases; 44 isolates producing OXA-23, 40, 48, 58, 72, 181, and 232 class D carbapenemases; and 6 isolates producing 2 carbapenemases. The latter comprised two isolates of *K*. *pneumoniae* that produced NDM + OXA-181 and NDM + OXA-232; two isolates of *E*. *cloacae* that produced KPC-18 + VIM-1; and two isolates of *A*. *baumannii* that produced NDM + OXA-23 and OXA-23 + OXA-40. The 67 carbapenemase-negative isolates (non-CPOs) included porin mutants and producers of ESBLs, AmpCs, K1, and broad spectrum β-lactamases. Classification potential was assessed for 205 CPOs that produced a single carbapenemase class. These comprised 185 isolates from the detection part of the study (88 class A, 56 class B, 41 class D) and an additional 10 class A (9 KPC, 1 SME), 7 class B (4 VIM, 3 NDM) and 3 OXA-48-like class D producers. Control strains were *K*. *pneumoniae* BAA 1705 (KPC), *E*. *coli* BAA 2452 (NDM-1), *A*. *baumannii* CDC 0035 (OXA-72), and *K*. *aerogenes* (formerly *E*. *aerogenes*) G1614 (non-CPO).

The resistance mechanisms of the individual isolates and problematic test results are provided in the S1 Table.

### Carbapenemase detection

The CPO Complete test did not require an initial lysis step prior to incubating the test. The test solution (solution A) was prepared by dissolving 12 mg of pharmaceutical imipenem/cilastatin (Hospira cat. no. NDC 0409-3507-21), 10 mg thimerosal (Enzo cat. no. ALX-400-013-G005), 5 mg glucose (Sigma cat no. G-5000) and 4 mg polymyxin B (EMD Millipore Corp., USA, cat. no. 5291-500MG) in a mixture of 1 ml of Mueller-Hinton broth (BD Diagnostics Systems, Sparks, MD), 30 μl zinc sulfate (Sigma-Aldrich Co. cat. no Z2876) and 140 μl phenol red solution (VWR International catalog # 97062–476). This solution was pH adjusted to pH 7.0 using 10N NaOH & 12N HCl.

**Fig 1:**
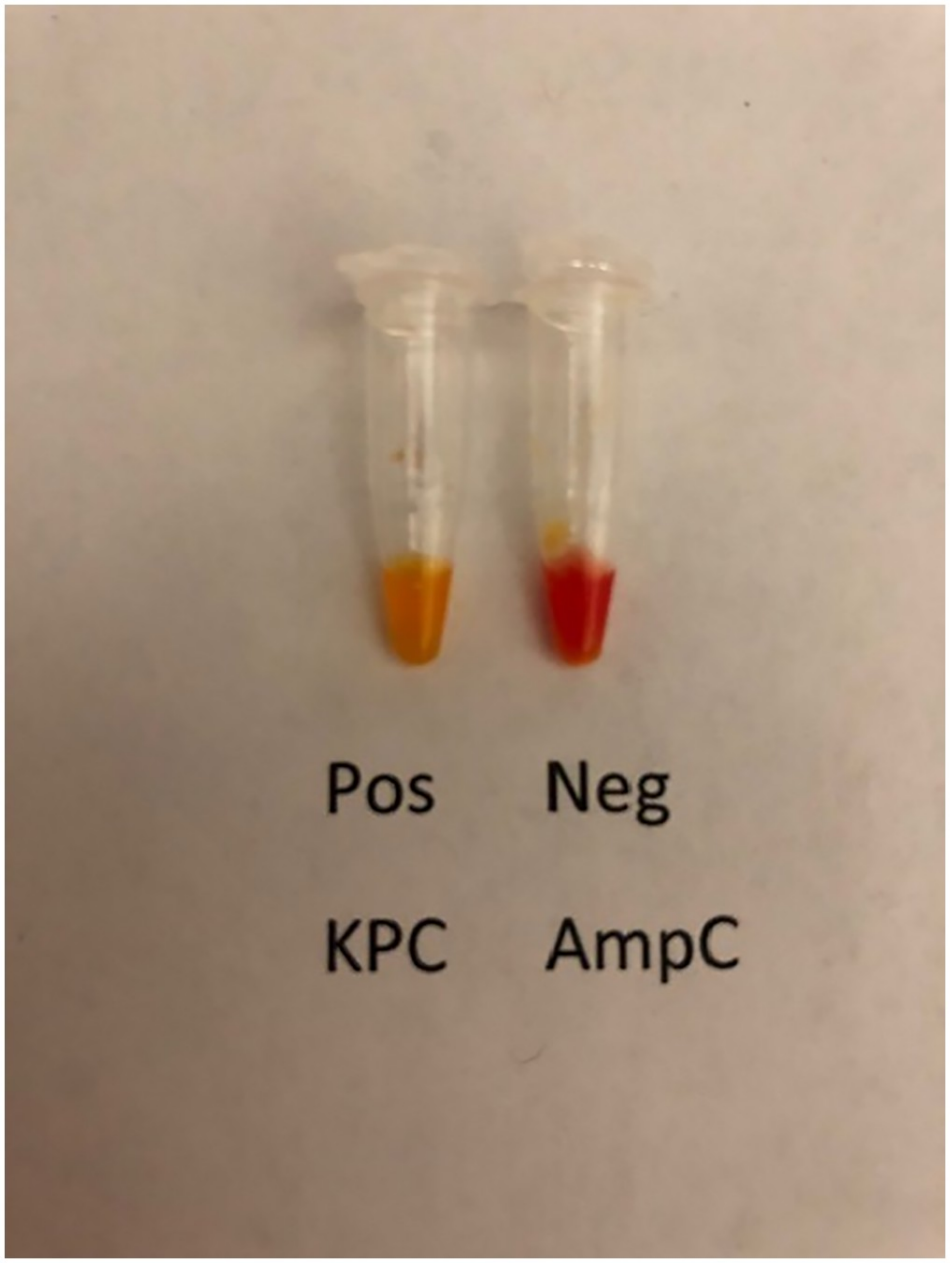
A positive test was interpreted as the development of yellow, orange or a lighter shade of red than the negative control. Tests with the positive control isolate, KPC-producing *K*. *pneumoniae* BAA 1705 and the negative control isolate *K*. *aerogenes* G1614 are shown.

Thirty μl of solution A was dispensed into transparent vessels such as PCR tubes (VWR International catalog # 20170–004) or microtiter wells. One tube was used for each test isolate and two additional tubes were used for testing a positive and negative control isolates. Colonies of each bacterial test isolate and a positive and negative control isolate were harvested with a 1 μl loop (VWR International, catalog # 12000–806) from blood agar (BD Diagnostics Systems, Sparks, MD, USA). The amount of harvested inoculum was sufficient to provide a slightly convex surface after filling the loop aperture, as opposed to a bulging loop with an excessive amount of inoculum. Excess inoculum was avoided as it may reduce test accuracy. The inoculum for each isolate was suspended in 30 μl of Solution A by vigorously rotating the loop in the solution. Inoculated tubes were then incubated at room temperature until positive or for a maximum of 90 minutes. The test was interpreted in bright light against a white background by comparing the color of the inoculated test to the negative control *K*. *aerogenes* G1614. A positive test was interpreted as the development of yellow, orange or a lighter shade of red than the red negative control (Fig 1). Tests were performed blinded.

### Carbapenemase classification

In contrast to the carbapenemase detection tests in which tests were performed blinded, the classification phase of the study was proof-of-concept testing with carbapenemase classifications known at the time of testing.

Three solutions were used for classification. These were solutions A (described above), B and C. Solution B contained phenyl boronic acid (VWR International catalog # BDH1115-1LP) to inhibit class A carbapenemases and solution C contained the chelating agents pyridine-2,6-dicarboxylic acid (Alfa Aesar cat no A12263) and Tris-EDTA buffer (Sigma T9285) to inhibit class B carbapenemases. Solution B comprised 30 μl of solution A supplemented with 2 μl of a solution prepared by dissolving 120 mg of phenyl boronic acid in 3 ml dimethyl sulfoxide and adding this to 3 ml sterile inoculum water (Beckman Coulter, Inc. Brea, CA, cat no B1015-2) and 840 μl phenol red solution. The final solution was pH adjusted to pH 7.5 using 10N NaOH and 12 N HCl. Solution C comprised 30 μl of solution A supplemented with 2 μl of a solution prepared by dissolving 235 mg pyridine-2,6-dicarboxylic acid, 98% (also known as dipicolinic acid) in 10 ml Tris-EDTA buffer solution 100x concentrate and 1,400 μl phenol red solution. The final solution was pH adjusted to pH 6.8 using 10 N NaOH and 12N HCl.

**Fig 2:**
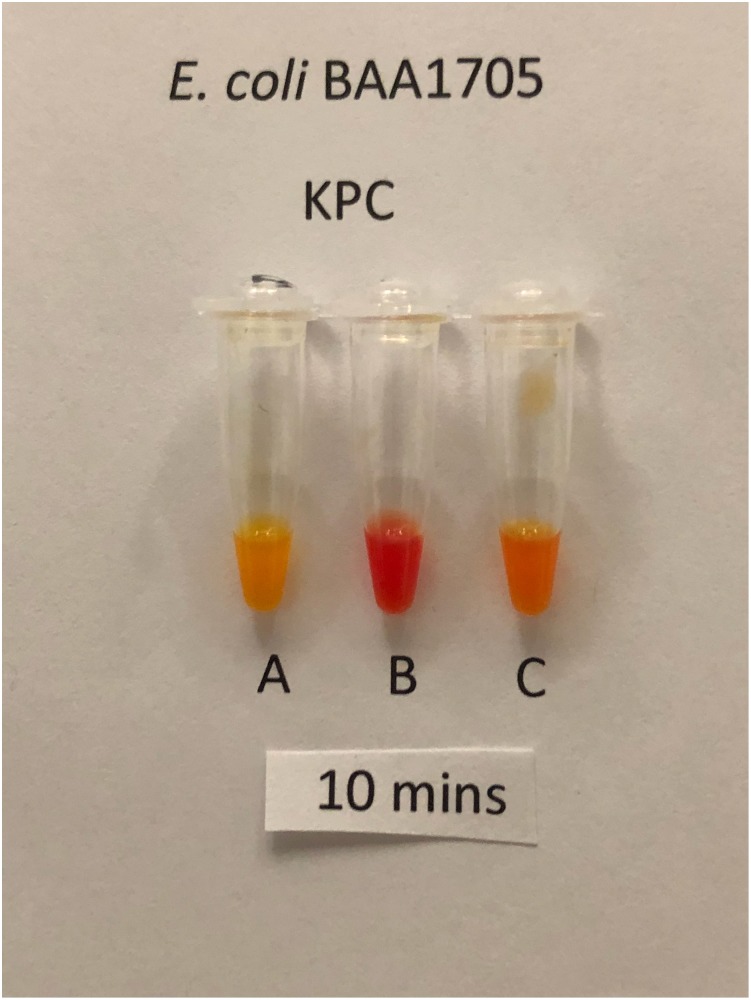
Classification test for Class A CPO, KPC-producing *K*. *pneumoniae* BAA 1705 after 10 minutes incubation. Tubes A and C are positive (yellow). Tube B is negative (red). Tube A contains only the test solution and indicates that the isolate is carbapenemase-positive. Tubes B and C contain the test solution plus inhibitors of Class A and B carbapenemases respectively.

**Fig 3:**
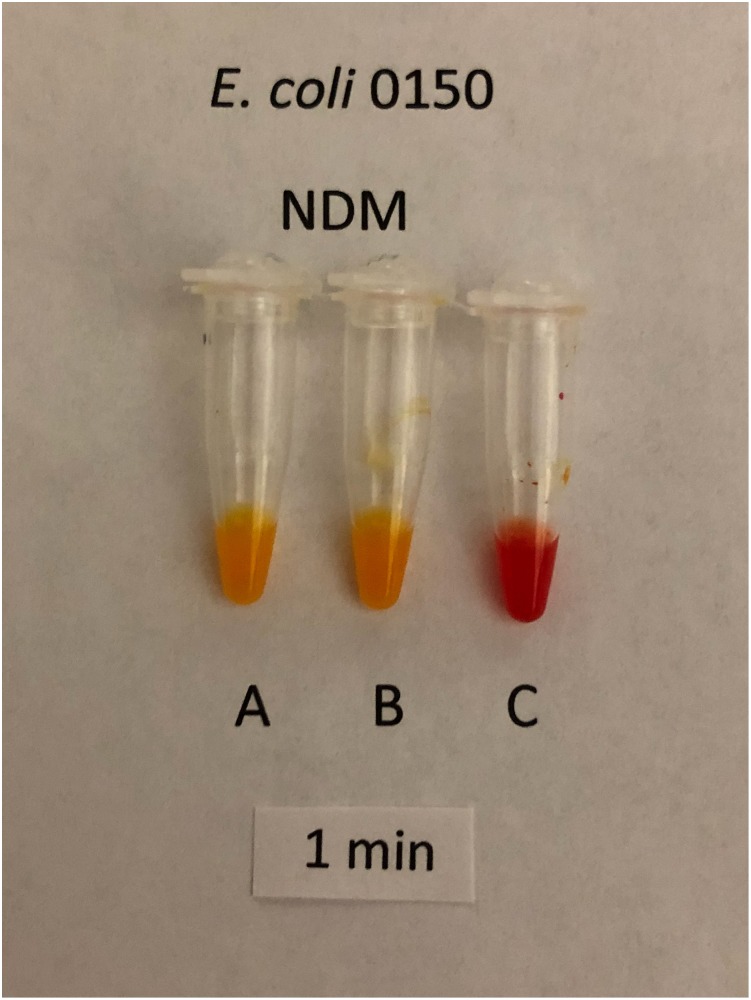
Classification test for Class B CPO, NDM-5-producing *E*. *coli* CDC 0150 after 1 minute incubation showing positive results in tubes A and B and a negative result in tube C.

**Fig 4:**
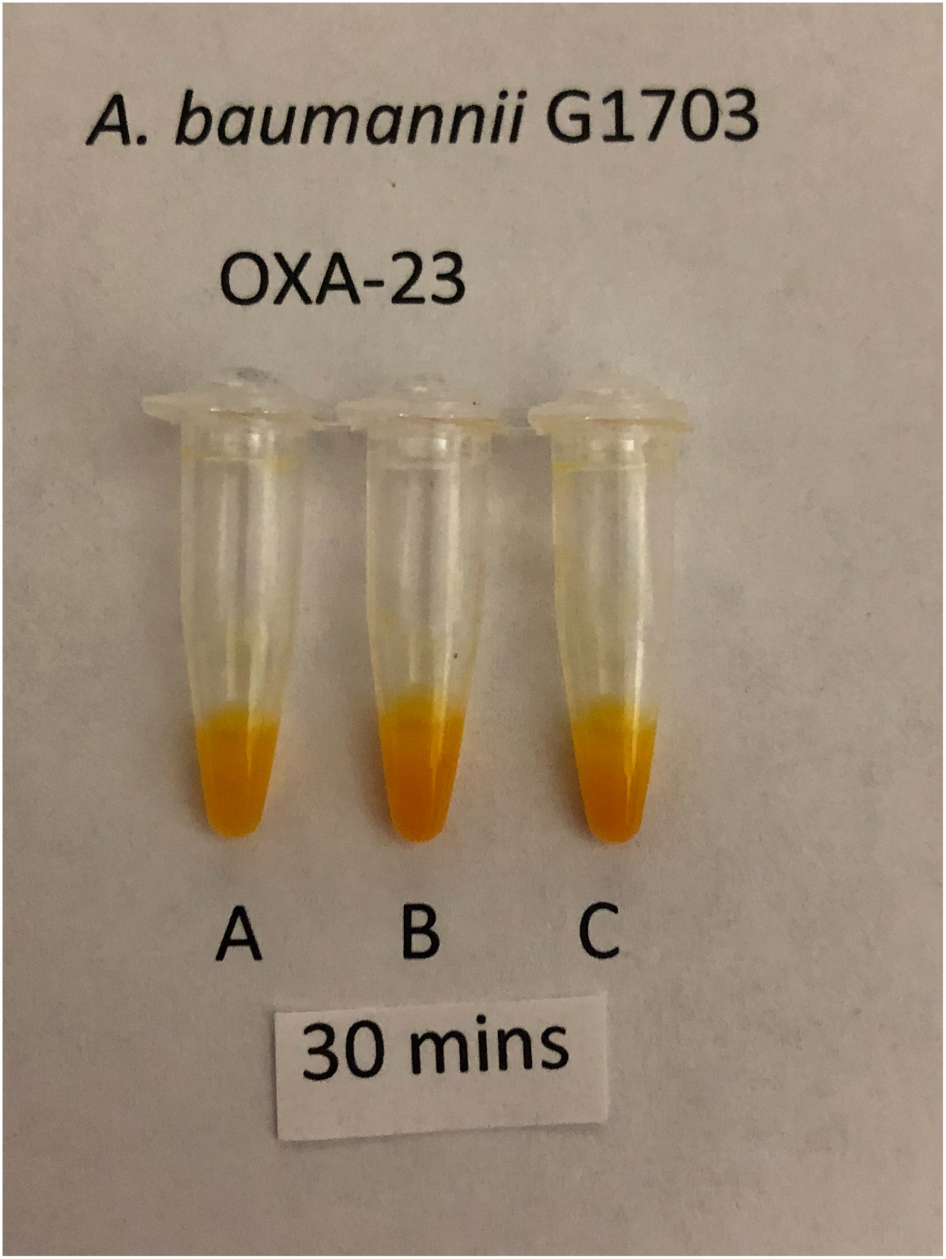
Classification test for Class D CPO, OXA-23-producing *A*. *baumannii* G1703 after 30 minutes incubation showing positive results in all three tubes.

Using a separate 1 μl loop for each tube, sets of the three solutions were inoculated for each of 205 isolates that produced a single carbapenemase. Tests with solutions B and C were inoculated by the same procedure used for tests with solution A. The isolates comprised 98 producers of class A carbapenemases, 63 producers of class B carbapenemases and 44 producers of class D carbapenemases. Only CPOs (i.e. positive result with solution A) were assessed for carbapenemase classification. Tests with solutions B and C scored as positive or negative by interpreting visually for a change from the initial red color to a lighter color. The interpretation was based on which of solutions B or C was more positive (i.e. lighter in color). Occasionally the color changes in solutions B or C were slower and less intense than the color change in solution A. Tests with solutions B and C were ignored if solution A yielded a negative result. Classifications were interpreted according to Table 1. Figs 2–4 show representative classification test results.

**Table 1:** Guide to Interpretation of carbapenemase classification tests.

Carbapenemase Classification	Solution		
	A	B	C
**Class A**	Positive	Negative	Positive
**Class B**	Positive	Positive	Negative
**Class D**	Positive	Positive	Positive
**Positive Untyped**	Positive	Negative	Negative
**Negative**	Negative	Not applicable	Not applicable

## Results

### Carbapenemase detection

All 262 CPOs yielded positive tests within 90 minutes (100% sensitivity) and only one of 67 non-CPOs yielded a falsely positive result (98.5% specificity) (Table 2). The carbapenemases of 74.1% of the CPOs were detected within seconds to 10 minutes and 98.1% were detected within 1 hour. Notably, KPC-producing *P*. *aeruginosa* isolates typically yielded positive results within 1 minute, 91.3% of isolates producing an OXA carbapenemase were positive within 60 minutes, and positive results were obtained with isolates of high diagnostic difficulty such as KPC-producing *A*. *baumannii*, KPC-4-producing *K*. *pneumoniae* and IMP-27-producing *Proteus mirabilis*. The single falsely positive result occurred with an AmpC-producing *E*. *cloacae* isolate.

**Table 2:** CPO detection and classification test results.

**CPO Detection**	**No. of Isolates**	**Positive**	**Negative**
**Class A**	125	125	0
**Class B**	87	87	0
**Class D**	44	44	0
**Dual Carbapenemases**	6	6	0
**All CPOs**	**262**	**262 (100%)**	**0 (0%)**
**Non-CPOs**	**67**	**1 (1.5%)**	**66 (98.5%)**
**Classification** **Single Carbapenemase CPOs**	**No. of Isolates**	**Correct**	**Incorrect or Unclassified**
**Class A**	98	98	0
**Class B**	63	63	0
**Class D**	44	42	2
**All**	**205**	**203 (99.0%)**	**2 (1.0%)**

### Carbapenemase classification

As shown in Table 2, 99% of isolates producing a single carbapenemase were correctly classified. All class A and B carbapenemases were classified correctly and 42 of the 44 class D carbapenemases were classified correctly. Two OXA-48-producing *K*. *pneumoniae* isolates yielded a positive but unclassified result.

Of the isolates that produced two carbapenemases, the three isolates that produced NDM plus a class D carbapenemase yielded a class B classification. The isolate that produced two class D carbapenemases yielded a class D classification, and the two isolates that produced KPC-18 + VIM-1 yielded a class D classification.

## Discussion

The high mortality and continuing emergence of resistance of CPOs make it essential that laboratories provide a strong diagnostic contribution in meeting the need to reduce the mortality and control the spread of these pathogens. Rapid, accurate CPO detection can facilitate prompt, appropriate, targeted therapy and effective infection control measures [24]. Detection tests that require overnight incubation are too slow for therapeutic and infection control needs but may be suitable for epidemiological studies [25, 26]. It is also important to use detection tests that minimize falsely positive results as these can have adverse consequences for patients such as being repeatedly subjected to unwarranted infection control precautions and receiving suboptimal, toxic therapy such as polymyxins [27].

It is now vitally important that laboratories rapidly classify carbapenemases to inform clinicians about the potential therapeutic utility of the new β-lactamase inhibitor combinations. Identification of class A carbapenemases indicates that the currently FDA-approved agents, ceftazidime/avibactam, meropenem/vaborbactam and imipenem/relebactam, are potential therapeutic options, while the detection of a class B carbapenemase contraindicates these agents. Classification can be a clinically powerful source of information about whether or not these agents are contenders for therapy but it does not eliminate the need for antibiotic susceptibility testing [16].

Carbapenemase classification has a second vital role in antibiotic stewardship for prevention of emergence of resistance to the new β-lactamase inhibitor combinations. These agents provide the opportunity to safely treat patients with CPO infections and avoid highly toxic agents such as the polymyxins and aminoglycosides. It is of critical importance to ensure that the currently approved new β-lactamase inhibitor combinations are not overused and select resistance, not only to themselves, but possibly also to the other β-lactamase inhibitor combinations currently in development. In particular, the potential for development of resistance to avibactam [28, 29] is a concern as this may impart cross-resistance to the related diazobicyclooctane analogs, relebactam and nacubactam. Similarly, resistance to vaborbactam may impart cross-resistance to its chemically related counterpart, VNRX-5133. Carbapenemase classification is a tool that can help to prevent overuse and guide appropriate use of the current new inhibitor combinations so that they are used almost solely for infections caused by class A CPOs and are restricted for infections by pathogens with other resistance mechanisms. This is vital because, despite the efficacy of these agents for class A CPO infections [30–34], gram-negative resistance continues to emerge [25, 35–40] and threatens our current window of opportunity for treating CPO infections with agents less toxic than those previously available.

A strength of this study was the variety of genotypes and phenotypes tested that included isolates of considerable diagnostic difficulty. Limitations were the non-blinded nature of the carbapenemase classification testing and the failure to include a complete range of types and levels of expression of carbapenemases and other β-lactamases. It is now necessary to determine the accuracy of classifications in blinded testing, to extend the range of carbapenemases and non-carbapenemases tested, and to test a larger collection of isolates that produce more than one class of carbapenemase. Based on the small sample of dual carbapenemase producers in this study it would be premature to make conclusions about one type of carbapenemase always being phenotypically dominant over another in classification tests. Phenotypic classification results for dual carbapenemase producers may depend not only on the enzyme classes but also on the relative amounts of activity of the co-produced enzymes. It was, nevertheless, a promising finding that for isolates producing a single carbapenemase CPO Complete correctly classified 99% of carbapenemases and that no class B CPOs were misclassified as class A.

In conclusion, in this study the CPO Complete test detected all carbapenemases rapidly, with a 10-minute detection rate of 74.1%. The speed and accuracy of the test, coupled with its potential to classify carbapenemases, and its applicability not only to *Enterobacteriaceae* and *P*. *aeruginosa* but also to *A*. *baumannii*, can be applied successfully to meeting what has become one of the world’s most urgent infectious disease challenges [3, 8, 14, 26, 41–49]. In addition to its accurate performance, the test has potential for incorporation in currently available automated susceptibility tests. Unlike molecular probes that may be able to detect only a small number of molecular targets and unable to distinguish between carbapenemase and non-carbapenemase *bla*_KPC_ variants, this and other phenotypic tests are likely to become increasingly useful as KPC variants continue to emerge [24, 40].

Furthermore, CPO Complete can be stored lyophilized at temperatures up to 38°C making it amenable to implementation in both limited resource and technologically advanced laboratories. In all, these features suggest that CPO Complete can contribute to the challenges of improving patient management, reducing CPO-associated mortality, and containing the spread of CPOs.

## Supporting information

S1 Table Characteristics of isolates and problematic results. Table provides details of individual isolates–identify, resistance mechanism(s), Ambler classification of carbapenemases, incorrect test results.
(PDF)
