# Supplementary material for: CPO Complete, a novel test for fast, accurate phenotypic detection and classification of carbapenemases
Source: PLoS One. 2019 Dec 11;14(12):e0220586. doi: 10.1371/journal.pone.0220586 (PMC6905549; doi:10.1371/journal.pone.0220586)
Supplement: S1 Table — Table provides details of individual isolates–identify, resistance mechanism(s), Ambler classification of carbapenemases, incorrect test results. (PDF) [file pone.0220586.s001.pdf]

S1 Table. **Characteristics of Isolates and Problematic Results**

Code	Organism	Mechanism(s)	Ambler Class of Carbapenemase	Incorrect Test Result(s)
0032	*E. cloacae*	KPC 3 TEM-1	A	
0061	*E. coli*	KPC-3 TEM-1	A	
0050	*E. cloacae*	KPC-low mic	A	
0053	*E. cloacae*	KPC-3 TEM-1	A	
0091	*S. marcescens*	SME	A	
0093	*E. cloacae*	KPC	A	
0096	*K. ozaenae*	KPC	A	
0097	*K. pneumoniae*	KPC	A	
0098	*K. pneumoniae*	KPC	A	
0104	*E. coli*	KPC-low mic	A	
0099	*S. marcescens*	SME	A	
0114	*E. coli*	KPC-3	A	
0116	*C. freundii*	KPC-2	A	
0132	*E. cloacae* group	IMI	A	
0133	*M. morganii*	KPC	A	
0134	*R. ornithinolytica*	KPC	A	
0144	*K. ascorbata*	KPC	A	
0155	*P. mirabilis*	KPC	A	
0164	*E. cloacae complex*	IMI	A	
G152	*E. cloacae*	KPC	A	
G153	*S. marcescens*	KPC	A	

G154	*K. pneumoniae*	KPC	A	
G1529	*E. cloacae*	KPC	A	
G15306	*E. cloacae*	KPC-2-like	A	
G15312	*E. cloacae*	KPC-2-like	A	
G15239	*E. coli*	KPC-3 like	A	
G156	*E. cloacae* or *asburiae*	KPC-2	A	
G15004	*K. oxytoca*	KPC	A	
G15346	*K. oxytoca*	KPC	A	
G157	*K. pneumoniae*	KPC-8	A	
G158	*K. pneumoniae*	KPC-8	A	
G159	*K. pneumoniae*	KPC-6	A	
G1510	*K. pneumoniae*	KPC-4	A	
G1511	*K. pneumoniae*	KPC-4	A	
G1513	*K. pneumoniae*	KPC-3 like	A	
G1514	*K. pneumoniae*	KPC-3,SHV-12-like,SHV-1, OXA-9-like,TEM-1-like	A	
G15313	*K. pneumoniae*	KPC-2-like	A	
G15007	*K. pneumoniae*	KPC-2-like	A	
G15081	*S. marcescens*	SME-like	A	
G15295	*E. cloacae*	KPC	A	
G1749	*C. freundii*	KPC	A	
G15314	*K. pneumoniae*	KPC-2-like	A	
G1640	*K. oxytoca*	KPC	A	
G15008	*K. pneumoniae*	KPC-2-like	A	

G15323	*K. pneumoniae*	KPC-2-like	A	
G15324	*K. pneumoniae*	KPC-2-like	A	
G15325	*K. pneumoniae*	KPC	A	
G15010	*K. pneumoniae*	KPC-2-like	A	
G15331	*K. pneumoniae*	KPC	A	
G15342	*K. pneumoniae*	KPC	A	
G15344	*K. pneumoniae*	KPC	A	
G15011	*K. pneumoniae*	KPC	A	
G15166	*S. marcescens*	SME-like	A	
G1690	*K. pneumoniae*	KPC	A	
G1705	*K. pneumoniae*	KPC	A	
G1650	*K. pneumoniae*	KPC	A	
G1675	*K. pneumoniae*	KPC-2, SHV-5-like,TEM-1-like	A	
G1706	*C. freundii*	KPC	A	
G1712	*E. cloacae*	KPC	A	
G1724	*C. amalonaticus*	KPC-2-like	A	
G1639	*K. pneumoniae*	KPC-2	A	
G1668	*K. pneumoniae*	KPC	A	
G1725	*K. pneumoniae*	KPC	A	
G1727	*K. pneumoniae*	KPC	A	
G1729	*K. pneumoniae*	KPC	A	
G15268	*E. cloacae*	KPC	A	
G1756	*K. pneumoniae*	KPC	A	
G15327	*C. freundii* complex	KPC-3 like	A	

G15238	*E. coli*	KPC-3 like	A	
G15317	*K. pneumoniae*	KPC-2-like	A	
G15318	*K. pneumoniae*	KPC	A	
G15319	*K. pneumoniae*	KPC-2-like	A	
G15320	*K. pneumoniae*	KPC	A	
G15009	*K. pneumoniae*	KPC-2-like	A	
G15270	*E. cloacae*	KPC	A	
G15271	*E. cloacae*	KPC	A	
G15296	*E. cloacae*	KPC	A	
G15301	*E. cloacae*	KPC	A	
G15003	*E. cloacae*	KPC	A	
G15315	*E. cloacae*	KPC-2-like	A	
G15333	*E. cloacae*	KPC-2-like	A	
G15311	*K. pneumoniae*	KPC	A	
G1815	*K. oxytoca*	KPC	A	
G1801	*K. pneumoniae*	KPC	A	
G1798	*K. oxytoca*	KPC	A	
G1799	*E. cloacae*	KPC	A	
G1800	*K. pneumoniae*	KPC	A	
G1789	*C. freundii*	KPC	A	
G1795	*E. cloacae*	KPC	A	
G1794	*S. marcescens*	SME	A	
G1791	*K. variicola*	KPC	A	
G1788	*E. coli*	KPC	A	

G1802	*K. pneumoniae*	KPC	A	
G1803	*C. freundii*	KPC	A	
G15383	*K. pneumoniae*	KPC-3	A	
G15012	*K. pneumoniae*	KPC	A	
G15322	*K. pneumoniae*	KPC	A	
G15360	*K. pneumoniae*	KPC-3	A	
G15361	*K. pneumoniae*	KPC-3	A	
G15371	*K. pneumoniae*	KPC-3	A	
G1726	*K. pneumoniae*	KPC	A	
G1720	*K. pneumoniae*	KPC	A	
G1718	*K. pneumoniae*	KPC	A	
G1719	*K. pneumoniae*	KPC	A	
G1714	*K. pneumoniae*	KPC-2-like	A	
G1715	*K. pneumoniae*	KPC-2-like	A	
G1716	*K. pneumoniae*	KPC-2	A	
G1717	*K. pneumoniae*	KPC	A	
G1804	*K. pneumoniae*	KPC	A	
G1805	*E. cloacae*	KPC	A	
G1644	*K. pneumoniae*	KPC-2	A	
G15286	*K. pneumoniae*	KPC-2-like	A	
G15287	*K. pneumoniae*	KPC	A	
G15006	*K. pneumoniae*	KPC-2-like	A	
G15005	*K. pneumoniae*	KPC-2-like	A	
G15293	*K. pneumoniae*	KPC	A	

G15288	*K. pneumoniae*	KPC-2-like	A	
G1713	*K. pneumoniae*	KPC-2-like	A	
G15162	*E. cloacae*	NMC-A	A	
0090	*P. aeruginosa*	KPC	A	
G1694	*A. baumannii*	KPC	A	
G15	*P. aeruginosa*	KPC-5	A	
G15357	*P. aeruginosa*	KPC-5	A	
G15013	*P. aeruginosa*	KPC	A	
0231	*P. aeruginosa*	KPC-5, OXA-2	A	
0038	*E. cloacae*	NDM	B	
0040	*K. pneumoniae*	VIM	B	
0041	*K. pneumoniae*	NDM	B	
0046	*K. pneumoniae*	VIM	B	
0048	*E. coli*	NDM	B	
0049	*K. pneumoniae*	NDM	B	
0057	*M. morganii*	NDM	B	
0055	*E. coli*	NDM	B	
0063	*E. coli*	NDM	B	
0069	*E. coli*	NDM	B	
0076	*K. pneumoniae*	VIM	B	
0080	*K. pneumoniae*	IMP	B	
0082	*P. rettgeri*	NDM	B	
0118	*E. coli*	NDM	B	
0119	*E. coli*	NDM	B	

0127	*S. seftenberg*	NDM	B	
0128	*E. coli*	NDM	B	
0135	*K. pneumoniae*	VIM	B	
G15300	*K. pneumoniae*	IMP-8	B	
G15126	*S. marcescens*	IMP-1	B	
G15185	*P. mirabilis*	IMP-27	B	
G1691	*E. cloacae*	IMP-8	B	
G15291	*K. pneumoniae*	VIM-like,SHV-12-like,SHV-1-like	B	
G15408 (1)	*P. rettgeri*	NDM-1	B	
G15405	*E. cloacae*	NDM	B	
G15407	*S. marcescens*	NDM-1	B	
G15409	*K. pneumoniae*	NDM-1,CTX-M-15,TEM-1,OXA-1	B	
G15410	*K. pneumoniae*	NDM-1,CTX-M-15,CMY-16,TEM-1	B	
G15416	*C. freundii*	NDM-1	B	
G15417	*K. pneumoniae*	NDM-1	B	
G15418	*K. pneumoniae*	VIM-1	B	
G15353	*K. pneumoniae*	NDM-1,CTX-M-15-like,SHV-1-like	B	
G15403	*K. pneumoniae*	NDM	B	
G15355	*K. pneumoniae*	NDM-1,CTX-M-15-like,SHV-1-like	B	
G15404	*E. cloacae*	NDM	B	
G15411	*K. pneumoniae*	NDM-1	B	
G15412	*E. coli*	NDM-1,CTX-M-15	B	
G15413	*E. coli*	NDM-1	B	
G15414	*E. coli*	NDM-1,CTX-M-15,TEM-1	B	

G15415	*C. freundii*	NDM-1	B	
G1746	*C. freundii*	NDM-1,CTX-M-15,TEM-1,OXA-1	B	
G1757	*E. coli*	NDM-1	B	
G15406	*K. pneumoniae*	NDM-1,CTX-M-15,OXA-181	B	
0034	*K. pneumoniae*	IMP	B	
0106	*K. pneumoniae*	NDM	B	
G1793	*E. cloacae*	VIM	B	
G1819	*C. koseri*	VIM	B	
0054	*P. aeruginosa*	VIM-4	B	
0064	*P. aeruginosa*	SPM	B	
0088	*A. baumannii*	NDM-1	B	
0092	*P. aeruginosa*	IMP-14	B	
0100	*P. aeruginosa*	VIM-2	B	
0103	*P. aeruginosa*	IMP-1	B	
0108	*P. aeruginosa*	VIM	B	
0110	*P. aeruginosa*	VIM	B	
0111	*P. aeruginosa*	VIM	B	
0033	*A. baumannii*	NDM-1	B	
0037	*A. baumannii*	NDM-1	B	
G15303	*P. aeruginosa*	GIM-1	B	
G15304	*P. aeruginosa*	SPM-1	B	
G15236	*A. baumannii*	VIM-2	B	
G15297	*P. aeruginosa*	IMP-18	B	
G15015	*P. aeruginosa*	IMP-7	B	

G15401	*P. aeruginosa*	IMP-7	B	
G1692	*A. baumannii*	IMP-4	B	
G15021	*P. aeruginosa*	VIM-like	B	
G15557	*P. aeruginosa*	VIM-like	B	
G15402	*P. aeruginosa*	IMP-7	B	
G15017	*P. aeruginosa*	VIM-2	B	
G15019	*P. aeruginosa*	VIM-2	B	
G1708	*A. baumannii*	VIM-2	B	
G1709	*A. baumannii*	IMP-1	B	
G1651	*P. aeruginosa*	VIM-3	B	
0250	*P. aeruginosa*	NDM	B	
0254	*P. aeruginosa*	VIM-2	B	
0248	*P. aeruginosa*	VIM	B	
0249	*P. aeruginosa*	VIM	B	
0255	*P. aeruginosa*	VIM-2	B	
0245	*P. aeruginosa*	VIM	B	
0246	*P. aeruginosa*	VIM	B	
0239	*P. aeruginosa*	VIM-1,GES-1,OXA-10	B	
0240	*P. aeruginosa*	VIM	B	
0230	*P. aeruginosa*	VIM-2	B	
0241	*P. aeruginosa*	IMP	B	
0242	*P. aeruginosa*	VIM	B	
G15419	*P. aeruginosa*	VIM-2	B	
G15420	*P. aeruginosa*	VIM-2	B	

0039	*K. pneumoniae*	OXA-181	D	
0051	*K. ozaenae*	OXA-181	D	
0066	*K. pneumoniae*	OXA-232	D	
0074	*E. aerogenes*	OXA-48	D	
0075	*K. pneumoniae*	OXA-232	D	
0141	*K. pneumoniae*	OXA-181	D	
0142	*K. pneumoniae*	OXA-181	D	
G15481	*K. pneumoniae*	OXA-48	D	
G15483	*K. pneumoniae*	OXA-48	D	
G15482	*K. pneumoniae*	OXA-48	D	
G15486	*K. pneumoniae*	OXA-48	D	
G15490	*K. pneumoniae*	OXA-48	D	Unclassified
G15485	*K. pneumoniae*	OXA-48	D	
G15484	*K. pneumoniae*	OXA-48	D	
G15491	*K. pneumoniae*	OXA-181	D	
G15488	*K. pneumoniae*	OXA-48	D	
G15487	*K. pneumoniae*	OXA-48	D	
G15480	*K. pneumoniae*	OXA-48	D	
G15489	*K. pneumoniae*	OXA-48	D	Unclassified
0045	*A. baumannii*	OXA-23	D	
0052	*A. baumannii*	OXA-58	D	
0056	*A. baumannii*	OXA -23	D	
0035	*A. baumannii*	OXA-72	D	
0070	*A. baumannii*	OXA 58	D	

G1670	*A. baumannii*	OXA-23	D	
G1744	*A. baumannii*	OXA-40	D	
G1732	*A. baumannii*	OXA-23	D	
G1679	*A. baumannii*	OXA-23	D	
G1734	*A. baumannii*	OXA-40	D	
G1674	*A. baumannii*	OXA-23	D	
G1687	*A. baumannii*	OXA-23	D	
G1703	*A. baumannii*	OXA-23	D	
0036	*A. baumannii*	OXA-40	D	
273	*A. baumannii*	OXA-23	D	
282	*A. baumannii*	OXA-23	D	
284	*A. baumannii*	OXA-40	D	
290	*A. baumannii*	OXA-23	D	
291	*A. baumannii*	OXA-23	D	
303	*A. baumannii*	OXA-23	D	
306	*A. baumannii*	OXA-40	D	
309	*A. baumannii*	OXA-23	D	
311	*A. baumannii*	OXA-23	D	
313	*A. baumannii*	OXA-23	D	
G1733	*A. baumannii*	OXA-23	D	
0068	*K. pneumoniae*	NDM, OXA-181	B + D	B
0083	*A. baumannii*	NDM, OXA-23	B + D	B
0153	*K. pneumoniae*	NDM, OXA-232	B + D	B
G6809	*E. cloacae*	KPC-18, VIM-1	A + B	D

G6810	*E. cloacae*	KPC-18, VIM-1	A + B	D
0063	*A. baumannii*	OXA-23, OXA-40	D	
0042	*K. pneumoniae*	CTX-M-28, OMPK36, OMPK-35	Non-CPO	
0043	*K. pneumoniae*	SHV-12, OMPK-36	Non-CPO	
0044	*K. pneumoniae*	TEM-1, SHV-1, CTX-M-15	Non-CPO	
0062	*E. aerogenes*	cAmpC	Non-CPO	
0047	*K. pneumoniae*	OmpK35	Non-CPO	
0058	*E. coli*	ESBL	Non-CPO	
0060	*E. cloacae*	cAmpC	Non-CPO	
0065	*E. cloacae*	cAmpC	Non-CPO	Positive
0072	*E. cloacae*	cAmpC	Non-CPO	
0073	*E. cloacae*	cAmpC	Non-CPO	
0079	*K. pneumoniae*	CTX-M14; DHA-1 OMPK35	Non-CPO	
0081	*E. coli*	CMY-2 type	Non-CPO	
0086	*E. coli*	CTX-M-9	Non-CPO	
0087	*K. pneumoniae*	SHV	Non-CPO	
0089	*E. coli*	CMY-2 type	Non-CPO	
0107	*K. pneumoniae*	CTX-M-2, OMPK-36	Non-CPO	
0109	*K. pneumoniae*	TEM-1, SHV-1, CTX-M-15	Non-CPO	
G1743	*E. coli*	CTX-M-12	Non-CPO	
G1693	*E. coli*	IMI-R, high AmpC	Non-CPO	
G1615	*E. coli*	cAmpC	Non-CPO	
G1634	*E. coli*	High AmpC,TEM-1-like	Non-CPO	
G1700	*E. coli*	High AmpC,TEM-1-like	Non-CPO	

G1614	*E. aerogenes*	High AmpC	Non-CPO	
G1638	*E. aerogenes*	High AmpC, SHV-5-like	Non-CPO	
G1758	*K. pneumoniae*	CLSI MHT negative control	Non-CPO	
G1759	*K. pneumoniae*	SHV-18	Non-CPO	
G1753	*E. cloacae*	High AmpC	Non-CPO	
G1641	*E. coli*	High AmpC	Non-CPO	
G1683	*E. coli*	DHA-like	Non-CPO	
G1649	*S. marcescens*	SHV-7	Non-CPO	
G162	*K. pneumoniae*	FOX-5, SHV-4 like	Non-CPO	
G1735	*E. cloacae*	High AmpC	Non-CPO	
G1754	*K. pneumoniae*	TEM-1,SHV-1, OXA-9, SHV-5 ACT-1	Non-CPO	
G1685	*K. pneumoniae*	CMY-2-like	Non-CPO	
G1673	*K. pneumoniae*	CMY-2, SHV-1, TEM-1-like	Non-CPO	
G1702	*K. pneumoniae*	FOX-1	Non-CPO	
G164	*E. coli*	High AmpC, SHV-12 like	Non-CPO	
G1616	*K. pneumoniae*	LAT-4	Non-CPO	
G163	*K. oxytoca*	DHA-like, SHV ESBL, TEM-1-like, K1	Non-CPO	
G1751	*M. morganii*	High AmpC	Non-CPO	
G1745	*P. mirabilis*	CMY-like, TEM-1-like	Non-CPO	
G1747	*E. coli*	CMY-2	Non-CPO	
G1792	*E. coli*	CTX-M	Non-CPO	
G1796	*K. pneumoniae*	CTX-M	Non-CPO	
G1797	*E. coli*	TEM-10, TEM-12	Non-CPO	
G1701	*K. pneumoniae*	MOX-1	Non-CPO	

G1618	*P. aeruginosa*	OXA-6	Non-CPO	
G1619	*P. aeruginosa*	OXA-11	Non-CPO	
G16350	*P. aeruginosa*	Overexpressed MexAB-OprM, high AmpC	Non-CPO	
G16291	*P. aeruginosa*	Overexpressed MexXY, OprD diminished, high AmpC	Non-CPO	
G16368	*P. aeruginosa*	Overexpressed MexAB-OprM, OprD diminished	Non-CPO	
G1689	*K. oxytoca*	Low K1, TEM-1 like	Non-CPO	
TIM16	*K. pneumoniae*	SHV-1	Non-CPO	
TIM30	*K. pneumoniae*	High SHV-1	Non-CPO	
G1688	*K. pneumoniae*	TEM-1	Non-CPO	
G1665	*S. marcescens*	Wild type	Non-CPO	
G1660	*K. pneumoniae*	TEM-16	Non-CPO	
G1812	*P. aeruginosa*	Wildtype	Non-CPO	
G1778	*P. aeruginosa*	Partially derepressed	Non-CPO	
G1779	*E. coli*	TEM-2	Non-CPO	
G16369	*P. aeruginosa*	Overexpressed MexEF-OprN, OprD diminished	Non-CPO	
G16352	*P. aeruginosa*	Overexpressed MexAB-OprM, OprD diminished, high AmpC	Non-CPO	
G1782	*P. aeruginosa*	Wild type	Non-CPO	
G1672	*C. freundii*	Wild type + TEM-1	Non-CPO	
G16288	*P. aeruginosa*	Overexpressed MexXY, OprD not functional	Non-CPO	
G1686	*K. pneumoniae*	DHA-1	Non-CPO	

G1752	*P. aeruginosa*	High AmpC	Non-CPO	
